# Treatment for the Benign Childhood Epilepsy With Centrotemporal Spikes: A Monocentric Study

**DOI:** 10.3389/fneur.2021.670958

**Published:** 2021-05-06

**Authors:** Miriam Kessi, Fangling Yan, Langui Pan, Baiyu Chen, Eleonore Olatoutou, Dong Li, Fang He, Tibera Rugambwa, Lifen Yang, Jing Peng, Fei Yin

**Affiliations:** ^1^Department of Pediatrics, Xiangya Hospital, Central South University, Changsha, China; ^2^Hunan Intellectual and Developmental Disabilities Research Center, Changsha, China; ^3^Department of Oncology, Xiangya Hospital, Central South University, Changsha, China

**Keywords:** benign childhood epilepsy with centrotemporal spikes, intellectual disability, cognition, levetiracetam, sodium valporate, treatment

## Abstract

**Background and Purpose:** To date, there is no specific treatment guideline for the benign childhood epilepsy with centrotemporal spikes (BECTS). Several countries recommend levetiracetam, carbamazepine, sodium valproate, oxcarbazepine, and lamotrigine as first-line drugs. Nevertheless, some of these drugs are associated with cognitive decline. Available studies that investigated the efficacy of levetiracetam and sodium valproate on BECTS involved small sample sizes. This study aimed to evaluate the efficacy of levetiracetam and sodium valproate on cognition, and to investigate the prognostic factors for BECTS as whole.

**Methods:** Clinical data and treatment status of all patients with BECTS at Xiangya Hospital, Central South University followed from 2008 to 2013 were analyzed retrospectively. Since electrical status epilepticus in sleep (ESES) has been confirmed to play a role in cognitive deterioration, in order to evaluate the response to drugs and their cognitive effects, we created two groups of patients according to the levels of spike wave index (SWI): group 1; 0–50% SWI and group 2; >50% SWI at the last follow up.

**Results:** A total of 195 cases were enrolled: 49.7% received monotherapies, 24.1% duotherapies and 27.2% polytherapies. Medications included; levetiracetam plus other drug (s) (75.9%), levetiracetam alone (32.8%), sodium valproate plus other drug (s) (31.3%), and sodium valproate alone (5.1%). After 2 years of treatment and follow up, 71% of the cases had a good seizure outcome, 15.9% had an improvement of SWI, and 91.7% had a normal DQ/IQ. Sodium valproate combined with levetiracetam, and sodium valproate alone correlated with good improvement of SWI, whereas, focal spikes were linked with poor improvement. For both groups (group 1 and group 2): monotherapy, levetiracetam alone, and a normal DQ/IQ at seizure onset correlated with good cognitive outcomes, in contrast, polytherapy, sodium valproate plus other drug (s), levetiracetam plus sodium valproate, an initial SWI of ≥85%, and multifocal spikes were linked to cognitive deterioration.

**Conclusions:** Monotherapy, particularly levetiracetam seems to be a good first-line therapy which can help in normalizing the electroencephalograph and preventing cognitive decline. Polytherapy, mostly the administration of sodium valproate seems to relate with poor cognition, therefore, it is recommended to avoid it.

## Introduction

Benign childhood epilepsy with centrotemporal spikes (BECTS) forms a mild end of epilepsy aphasia spectrum (EAS), and is characterized by normal cognitive function but some cases can present with neuropsychological impairment, problems with cognition and academics (1, 2). It affects 15–25% of children below 15 years with a male predominance (2). BECTS can evolve to an atypical form known as atypical benign partial epilepsy of childhood (ABPE). ABPE is characterized by an earlier age of onset of seizures which are more severe such as atonic seizures, epileptic negative myoclonus, typical rolandic seizures (1), diurnal seizures, persistent seizures or status epilepticus, electrical status epilepticus in sleep (ESES) pattern in an electroencephalograph (EEG) and neuropsychological impairments (2). Neuropsychological impairments include learning/cognitive, and behavioral problems (3–5) as well as language deficits (6). Early age at epilepsy onset, presence of new seizures, and an increased frequency of spikes in EEG during sleep and daytime can predict evolvement of BECTS to ABPE (2). Neurocognitive deficits have been correlated with ESES as it is considered as a factor that negatively affects the cognitive aspects precisely because it interferes with the cognitive functions of sleep including memory-learning process (2, 7).

In addition to ESES, some drugs can accelerate cognitive decline. Most cases with BECTS have rare seizures which can be controlled by one antiepileptic drug, however, there are some cases who need more than one drug especially those with early onset (below 4 years) (2). In one clinical trial, 10.6% of the cases received multidrug therapy and the existence of bilateral abnormalities in EEG was a predictor for the need of multidrug therapies (8). Several countries recommend the utilization of levetiracetam, carbamazepine, sodium valproate, oxcarbazepine, and lamotrigine as first-line drugs (9). Levetiracetam is more preferred due to its efficacy (in seizure control, EEG normalization, cognition, speech, and behavior) and less side effects (10–14). Moreover, as monotherapy, it has been shown to be effective even in other idiopathic generalized epilepsy, such as in the absence epilepsy and juvenile myoclonic epilepsy (15). Among those preferred first-line drugs, exposure to high dose of sodium valproate has been shown to associate with poor cognition (16) and has many other adverse effects (17). Early recognition of BECTS and mitigation of ESES with therapies that do not interfere with cognition might lower the rate of neuropsychological impairments. Currently, the available studies that investigated the efficacy of levetiracetam and sodium valproate on BECTS involved small sample sizes (11–14, 18–24).

Thus, in order to prevent further cognitive deterioration for the cases with BECTS, this study aimed to evaluate the effects of levetiracetam, sodium valproate and other drugs on cognition for 195 cases followed from 2008 to 2013. Additionally, treatment outcomes and prognostic factors will be discussed. To the best of our knowledge, this study has the largest sample size on the aspect of evaluating the efficacy of levetiracetam and sodium valproate on cognition. This study will aid clinicians in management issues.

## Materials and Methods

### Ethical Clearance

This study was reviewed and approved by the Institutional Ethics Committee of Xiangya Hospital Central South University, thus complying with the treaty agreed to in 1964 in Helsinki by the World Medical Association on ethical principles of human research for medical purposes and subsequent revisions of the same (2013). Both informed and written consents were obtained from the parents and/or legal guardians for study participation.

### Study Design and Participants

BECTS cases attended at the department of pediatric neurology, Xiangya Hospital, Central South University followed from 2008 to 2013 were recruited retrospectively. We included those who met the clinical and electroencephalographic diagnostic criteria for (1) BECTS, (2) ABPE, and (3) follow-up period of at least 2 years. The diagnosis of BECTS was done according to the International League against Epilepsy (ILAE) criteria (25). The exclusion criteria included cases with: (1) continuous spike-and-wave during sleep (ECSWS), (2) Landau-Kleffner syndrome (LKS), (3) focal epilepsies with secondary bilateral synchronies not fulfilling the criteria for BECTS, and (4) with follow up period of <2 years. A thorough history, physical and neurological examinations, sleep and awake EEGs, and brain magnetic resonance images (MRIs) were performed for all cases.

The spike wave index (SWI) percentage was obtained as the total number of minutes of all spike and slow wave abnormalities divided by the total number of minutes of non-rapid eye movement sleep (NREM) and multiplied by 100. The follow-up EEG examinations at 3 months, 6 months, 1 year and 2 years of the treatment were evaluated. The ranges of SWI considered were: typical ESES (85–100%) and atypical ESES (<85%).

The EEG response to treatment was summarized as follows: (1) normalization of the record, (2) improvement of ≥50% of SWI, (3) improvement of <50% of SWI, and (4) no improvement. The clinical responsiveness to therapy (ies) was categorized as the following: complete disappearance of clinical manifestations observed at the time of diagnosis of equal or more than 50%, and <50%. Follow-up EEGs under different treatment regimens were evaluated along with cognitive changes. Clinicians and parents/guardians provided feedback regarding the cognitive deterioration or improvement. The judgement about the improvement or deterioration of cognition was done by senior neurologists. A relapse was defined as a reincrease of the SWI to half or more of what it was before treatment. A remission was considered for the cases without any relapse of seizures or ESES pattern in an EEG for more than a year.

In order to summarize the treatment outcomes after 2 years of follow-up, several terms were used. The term “good/positive seizures outcome” was considered for the cases who achieved seizure freedom plus those who had ≥50% seizure frequency reductions compared with the baseline frequencies. In contrast the term “poor/negative seizure outcome” was applied for the cases without seizures improvement plus those with <50% seizure frequency reductions. The term “good/positive improvement of SWI” was used for the cases with normalization of the EEG record, and those with improvement of ≥50% of SWI. The term “poor/negative improvement of SWI” was applied to all cases with improvement of <50% of SWI or no improvement of SWI or an increased SWI. The term “good/normal cognition” was considered for the cases with normal intelligence quotient (IQ)/developmental quotient (DQ) (IQ ≥ 70 plus DQ ≥ 85) while the term “poor/abnormal cognition” was used for the cases with abnormal DQ/IQ (IQ < 70 plus DQ < 85).

### Diagnosis and Management Protocol at Our Center

All cases received clinical assessments, EEG and MRI examinations. Antiepileptic drug particularly levetiracetam was given first incase patient presented with both seizures and ESES pattern. Benzodiazepines and/or steroids were given as add-on therapy (ies) for the cases with ESES worsening. However, this did not apply to all cases since patients were attended by different neurologists. Priority of the drug (s) prescribed changed with time based on the available updates from the literature at the point of diagnosis.

The diagnosis of intellectual disability (cognitive deficit) was done according to the diagnostic criteria of the DSM-5 for intellectual disabilities (Diagnostic and Statistical Manual of Mental Disorders, Fifth Edition, American Psychiatric Association, 2013). Standardized age-related rating scales, clinical interview and observations were used for the assessment of the adaptive functioning. But the diagnosis was often made based on clinical judgment, rather than on formal standardized assessments, especially for young patients (26). Standardized age-related rating scales that were used include: Gesell Developmental Schedules for patients younger than 2–4 years, Wechsler Preschool and Primary Scale of Intelligence-Fourth Edition (WPPSI-IV) for patients aged between 4 and 6 years, and Wechsler Intelligence Scale for Children-Fourth Edition (WISC-IV) for patients who were ≥6 years old. More details regarding assessment of intelligence can be found in our previous studies (27, 28).

### Clinical Data Collection and Review

Demographic data and clinical information such as sex, the age of onset of seizures and ESES, pre-natal, natal and post-natal histories, etiology, neurodevelopment, behavior, history of febrile seizures, family history of epilepsy, seizure semiology, frequency of seizures, the DQ/IQ at the diagnosis, the baseline SWI, and the therapy (ies) used were extracted from the hospital database. In addition, information regarding seizure frequency, SWI, IQ/DQ, cognitive status, behavioral changes, treatments and outcomes were collected at 3 months, 6 months, 1 year and 2 years. Therefore, all cases received at least 4 EEG examinations.

Individuals with insufficient medical records or lacked baseline assessments were excluded in this study. All data were reviewed by two neurologists. Furthermore, at least one neuro-radiologist reviewed the brain images.

### Grouping of the Patients

Since ESES has been confirmed to play a role in cognitive deterioration, in order to evaluate the response to drugs and their cognitive effects, we created two groups of patients according to the levels of SWI. Group 1 consisted of the cases with a record of 0–50% SWI at the last follow up. Group 2 consisted of the cases with a record of >50% SWI at the last follow up. We then investigated the relationship between the effects of drugs on cognition for each group.

### Statistical Analysis

All analysis were carried out via IBM SPSS Statistics 22.0 software (IBM, Armonk, NY, USA). We excluded the missing values in data analysis. Categorical data were summarized in the form of frequencies and proportions, and analyzed with the Chi-square test or Fisher's exact test where applicable. *P*-value of ≤ 0.05 was considered statistically significant.

## Results

### Demographic Characteristics of the Cohort

A total of 195 cases met the inclusion criteria. Males accounted for 50.8% (99/195) of the cases. The mean age of seizure onset was 6.50 ± 2.28 SD years (range = 0.17–12.83) while for ESES was 7.34 ± 2.39 years (range, 2–16). Impaired motor skills were observed in 1 (0.5%) case, abnormal behavior in 3 (1.5%) cases, impaired memory in 11(5.6%) cases, language problems in 4 (2.1%) cases, learning problems in 16 (8.2%) cases, and social problems in 1 (0.5%) case. Of the 181 cases with information regarding the initial DQ/IQ prior to treatment, 15 (8.3%) had abnormal findings (Supplementary Table 1).

### Seizure Semiology and Spike Wave Characteristics

Seizure semiologies included partial motor seizures (*n* = 18, 9.2%), tonic-clonic seizures (*n* = 85, 43.6%), complex partial seizures (*n* = 84, 43.1%), epileptic falls (*n* = 4, 2.1%), absence seizures (*n* = 4, 2.1%), and febrile seizures (*n* = 7, 3.6%). Of the 185 cases with information regarding the initial SWI, 19 (10.3%) had ≥85%, and the remained (*n* = 166, 89.7%) had <85%. For the 193 cases with details about epileptic discharges, findings were as follows; focal discharges were observed in 108 (56%) cases, multifocal discharges in 18 (9.3%), localized spikes in 161 (83.4%), and generalized spikes in 25 (13%). Out of the 194 cases with recorded origin of spikes, rolandic accounted for 138 (71.1%) cases, bilateral rolandic origin was noticed in 87 (44.8%), unilateral rolandic in 51 (26.3%), right rolandic in 26 (13.4%) and left rolandic in 25 (12.9%) (Supplementary Table 1).

### Treatment Strategies

**Figure 1:**
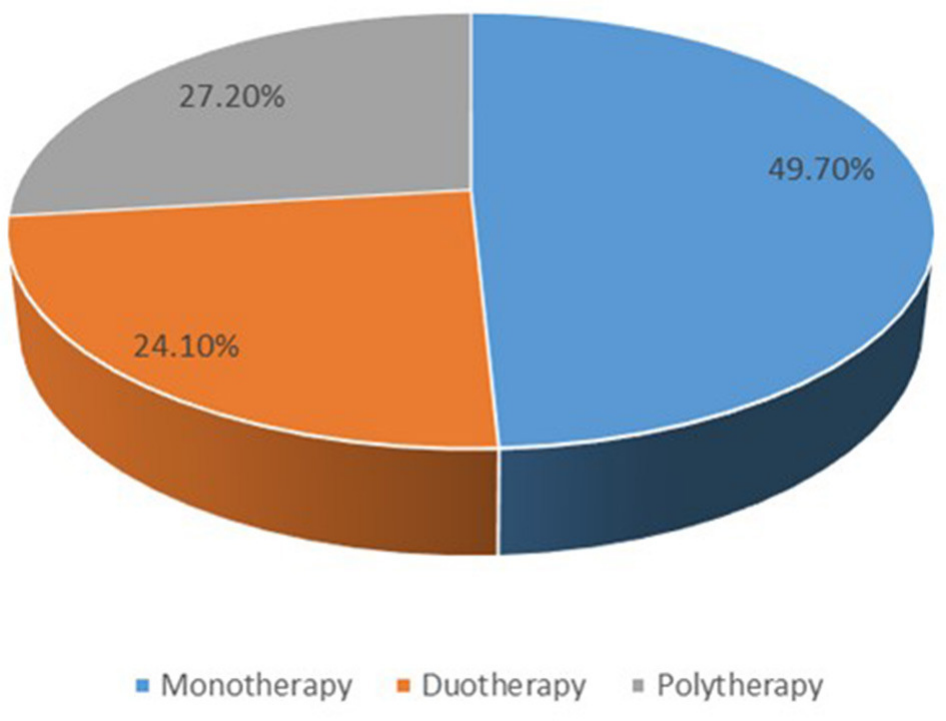
A summary of number of the therapies used.

**Figure 2:**
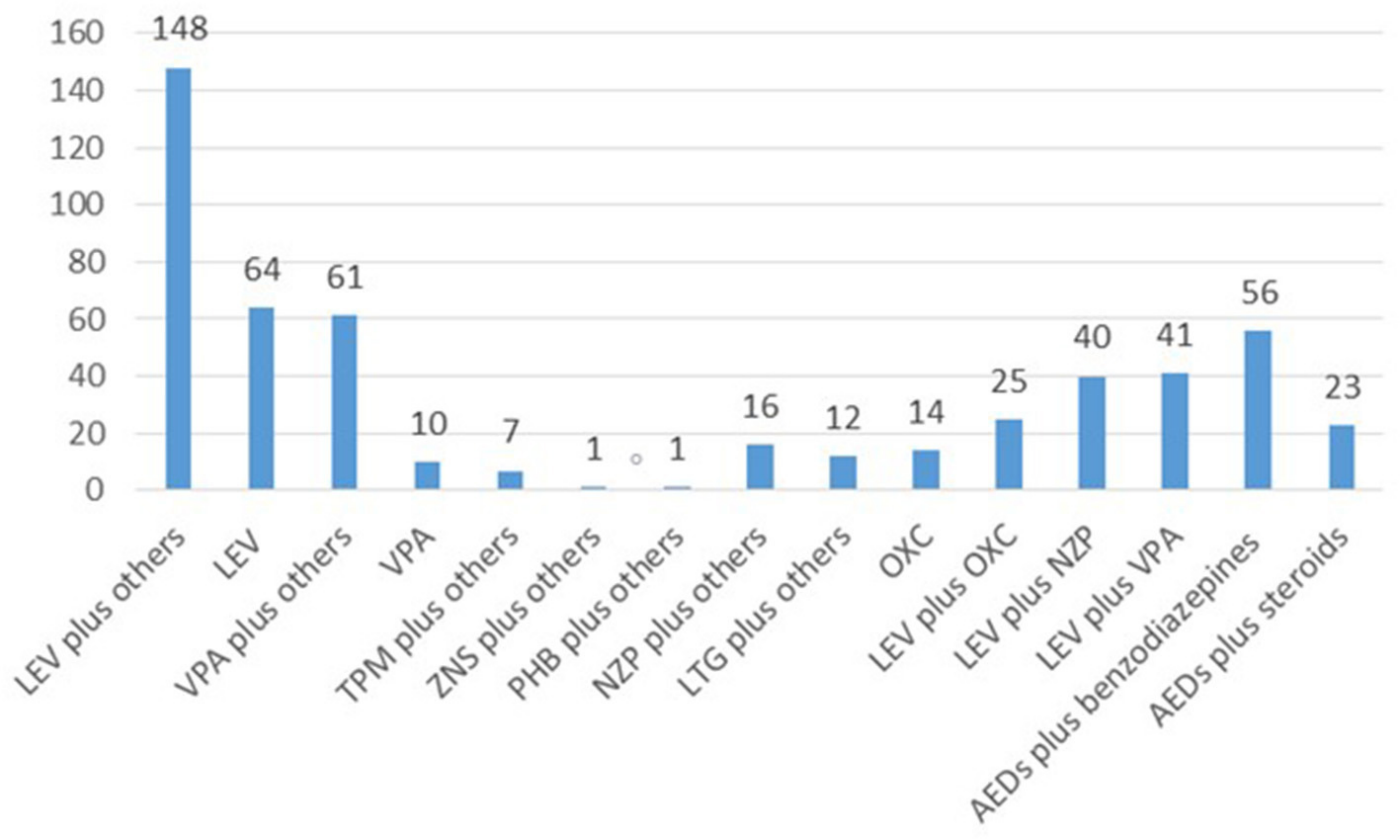
An overview of the specific drugs utilized in 195 cases. LEV, levetiracetam; VPA, sodium valproate; TPM, topiramate; ZNS, zonisamide; PHB, phenobarbital; NZP, nitrazepam; LTG, lamotrigine; OXC, oxcarbazepine.

Of the total number of patients, 97 (49.7%) received monotherapies, 47 (24.1%) duotherapies and 53 (27.2%) polytherapies (Figure 1). The therapies used were; levetiracetam plus other drug (s) (*n* = 148, 75.9%), levetiracetam alone (*n* = 64, 32.8%), sodium valproate plus other drug (s) (*n* = 61, 31.3%), sodium valproate alone (*n* = 10, 5.1%), oxcarbazepine alone (*n* = 14, 7.2%), topiramate plus other drug (s) (*n* = 7, 3.56%), zonisamide plus other drug (s) (*n* = 1, 0.5%), phenobarbital plus other drug (s) (*n* = 1, 0.5%), nitrazepam plus other drug (s) (*n* = 16, 8.2%), nitrazepam plus other drug (s) (*n* = 12, 6.2%), levetiracetam plus oxcarbazepine (*n* = 25, 12.8%), levetiracetam plus nitrazepam (*n* = 40, 20.5%), levetiracetam plus sodium valproate (*n* = 41, 21%), antiepileptic drugs plus benzodiazepines (*n* = 56, 28.7%), and antiepileptic drugs plus steroids (*n* = 24, 12.3%) (Figure 2).

### Outcome After 2 Years of Treatment and Follow Up

One hundred and eight six cases had information regarding seizures outcomes after 2 years of follow up; 116 (62.4%) cases became seizure free, 54 (29%) cases had ≥50% seizure frequency reductions compared with the baseline frequencies, and 28 (15.1%) cases had <50% seizure frequency reductions compared with the baseline frequencies or remained with the same frequency. Overall, 132 (71%) cases had good seizure outcomes (seizure free and ≥50% seizure frequency reductions compared with the baseline frequencies). Of the 182 cases with information regarding the final SWI, 21 (11.5%) had normalized EEGs, 82 (45.1%) had no EEG improvement or became worse, and 20 (11%) had increased SWIs. Overall, 29 (15.9%) cases had positive improvement of SWIs (normalized EEGs plus improvement of ≥50% of SWI). Of the 181 cases with information regarding the final DQ/IQ, 166 (91.7%) had normal DQ/IQ while 15 (8.3%) had abnormal DQ/IQ (Supplementary Table 1). Of those 15 cases. Five had normal baseline DQ/IQ of whom received sodium valproate plus oxcarbazepine (*n* = 1), levetiracetam only (*n* = 1), levetiracetam plus oxcarbazepine plus nitrazepam (*n* = 1), sodium valproate plus levetiracetam plus oxcarbazepine plus carbamazepine (*n* = 1), and sodium valproate plus levetiracetam plus nitrazepam plus lamotrigine (*n* = 1). At the last follow up, 61 cases had seizure remission, and 16 cases had EEG remission. The mean age of seizure remission was 11.28 ± 1.3 years (range, 10–14.83) while for ESES was 12.12 ± 2 years (range, 10–16).

### Determinants of Positive Seizure Outcome

There was no factor that showed correlation with good seizure outcome. However, sodium valproate (*P* = 0.066), epileptic falls (*P* = 0.074), speech delay (*P* = 0.074), and multifocal spikes (*P* = 0.090) showed non-statistically significant association with poor seizure outcome (Supplementary Table 2).

### Determinants of Positive Improvement of Spike Wave Index

Levetiracetam plus sodium valproate (*P* = 0.012), and sodium valproate alone (*P* = 0.030) showed correlation with positive improvement of SWI. Whereas, focal spikes (*P* = 0.025) and localized spikes (*P* = 0.031) were linked with poor improvement of SWI (Supplementary Table 3).

### Determinants of Good Cognitive Outcome After 2 Years of Treatment and Follow Up

#### Group 1

**Figure 3:**
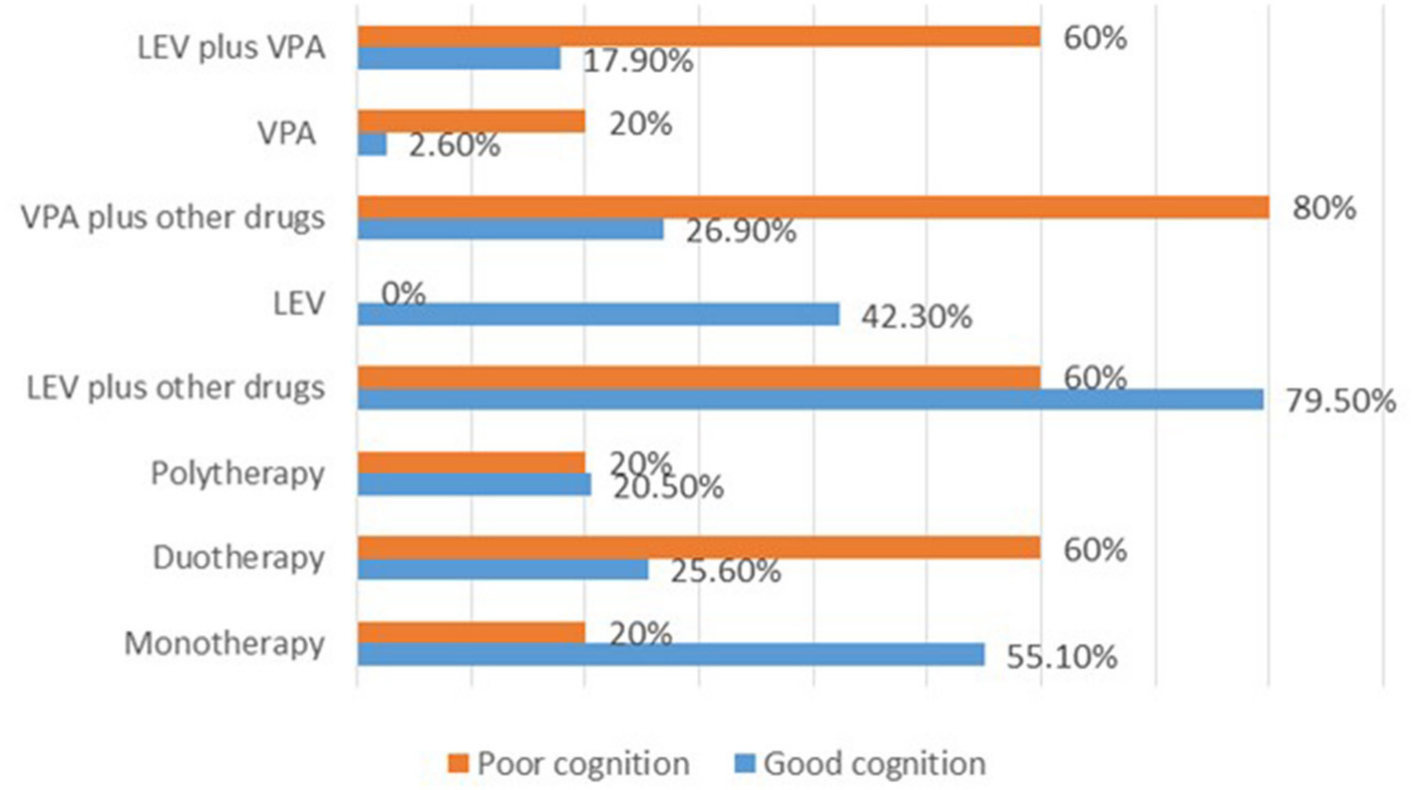
Summary of the therapies used and their effects on cognition after 2 years of treatment for the cases with 0–50% SWI at the last follow up. LEV, levetiracetam; VPA, sodium valproate.

For the 83 cases with recorded 0–50% SWI at the last follow up, normal DQ/IQ at seizure onset (*P* = 0.000) showed statistically significant association with good cognitive outcome. Whereas, the utilization of sodium valproate plus other drug (s) (*P* = 0.027) and levetiracetam plus sodium valproate (*P* = 0.056) showed an association with poor cognition (Supplementary Table 4). Besides, high proportion of cases with good cognition was observed for the cases that received monotherapies in contrast to those who received duotherapies or polytherapies. Notably, all cases that received levetiracetam alone (*n* = 33) had good cognitive outcome (Figure 3).

#### Group 2

**Figure 4:**
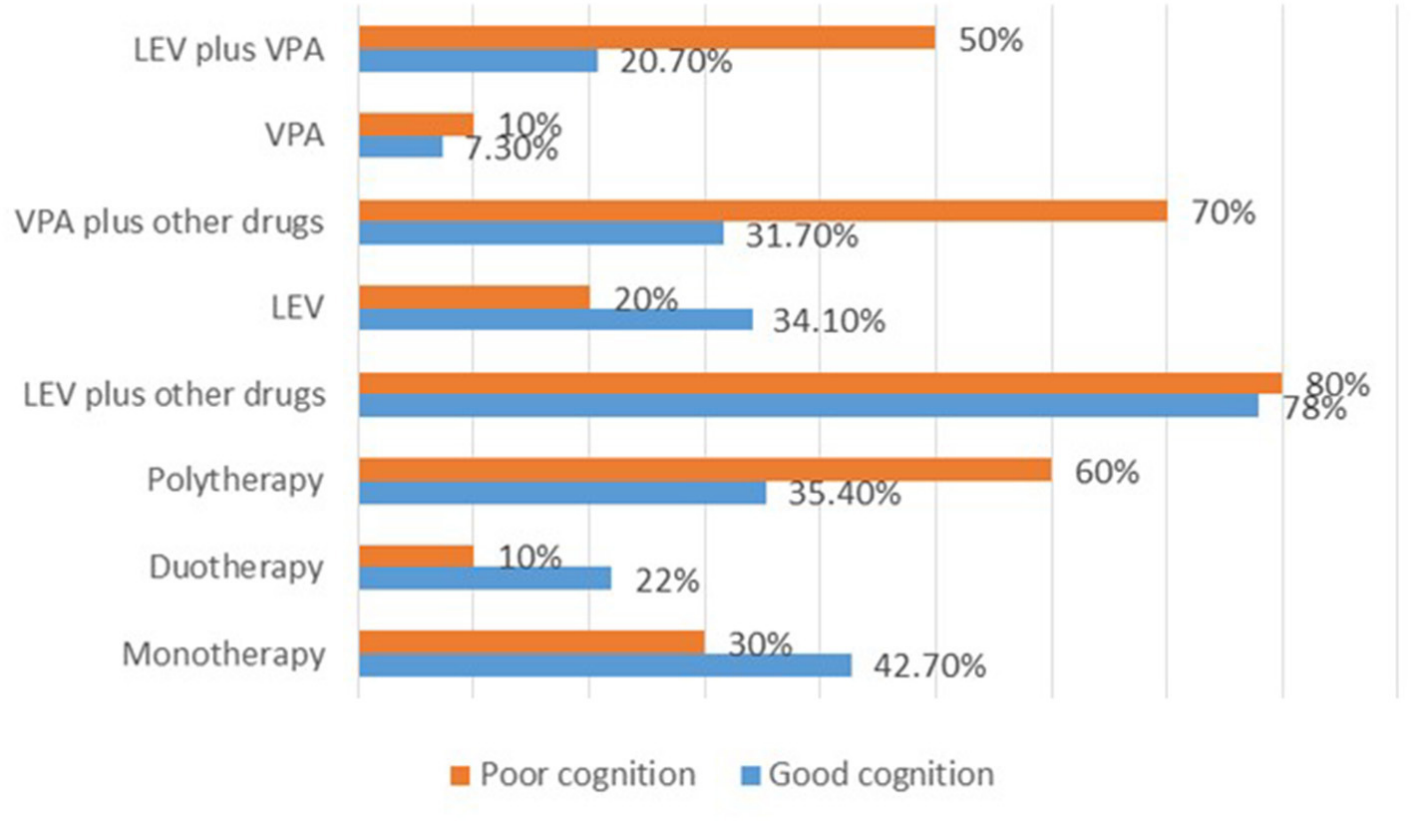
Summary of the therapies used and their effects on cognition after 2 years of treatment for the cases with >50% SWI at the last follow up. LEV, levetiracetam; VPA, sodium valproate.

For the 92 cases with recorded >50% SWI at the last follow up, normal DQ/IQ at seizure onset (*P* = 0.000), and ≥50% reduction of seizure frequency (*P* = 0.013) showed statistically significant association with good cognitive outcome. Whereas, initial SWI of ≥85% (*P* = 0.006), usage of sodium valproate plus other drug (s) (*P* = 0.032), utilization of levetiracetam plus sodium valproate (*P* = 0.055), and multifocal spikes (*P* = 0.096) showed correlated with poor cognitive outcome (Supplementary Table 5). Strikingly, high proportion of the cases with poor cognitive outcome was observed for those who received polytherapies (Figure 4).

## Discussion

In this study, the most common associated symptoms included learning problems, impaired memory and language problems. Abnormal baseline DQ/IQ prior to treatment accounted for 8.3% of the cases. Cases presented with variable seizure semiologies, and the commonest subtype was tonic-clonic. Approximately 90% of the cases had atypical ESES (<85% SWI). Almost 3/4 of the cases had spikes concentrated on rolandic region. Seventy one percent of the cases had good seizure outcome, and 15.9% had positive improvement of SWI. Similar to the baseline DQ/IQ prior to treatment, 8.3% of the cases had abnormal DQ/IQ after 2 years of treatment: 5 cases had normal findings before but transformed to abnormal after 2 years of treatment/follow up. The remained 10 cases had abnormal DQ/IQ from the beginning. Most cases received monotherapies, and the commonest prescribed drug was levetiracetam. The usage of levetiracetam plus sodium valproate as well as sodium valproate alone showed correlation with positive improvement of SWI, whereas, focal spikes and localized spikes were linked with negative improvement of SWI. For both groups (group 1 and group 2), monotherapy, the utilization of levetiracetam alone, and normal DQ/IQ at seizure onset seemed to relate with good cognitive outcome, whereas, polytherapy, utilization of sodium valproate plus other drug (s), levetiracetam plus sodium valproate, initial SWI of ≥85%, and the existence of multifocal spikes were linked to cognitive deterioration.

Most cases received monotherapies and polytherapies. The most commonly used drugs were levetiracetam alone or in combination with other drugs such as oxcarbazepine, sodium valproate and nitrazepam. Other countries recommend levetiracetam, carbamazepine, sodium valporate, oxcarbazepine, and lamotrigine as first-line drugs (9). Similar to our findings, levetiracetam is more preferred due to its efficacy (in seizure control, EEG normalization, cognition, speech, and behavior) and less side effects (10–14). In this study, levetiracetam plus sodium valproate showed statistically significant association with reduction of SWI. In comparison with carbamazepine and sodium valproate, levetiracetam was superior in suppressing rolandic discharges, however, the effects of those three drugs in controlling seizures were the same according to one study (29). A randomized controlled trial revealed that both levetiracetam and sulthiame have beneficial effects on EEG normalization (23). Besides, our study showed that the usage of sodium valproate alone correlates with poor seizure control. Nevertheless, one study showed sodium valproate and levetiracetam monotherapies are equally effective in controlling seizures, and sodium valproate was better than levetiracetam in normalizing EEG which is similar to our study (21).

Moreover, our study has shown that the utilization of the levetiracetam alone relates with good cognitive outcome which is similar to one study (18), however, it associates with poor cognitive outcome when administered along with sodium valproate. Exposure to high dose of sodium valproate has been shown to relate with poor cognition (16), and has many other adverse effects (17). Thus, based on findings from our study plus previous studies, we recommend levetiracetam as first-line therapy, and avoidance of sodium valproate whenever possible to prevent cognitive decline. Overall, 71% cases had good seizure outcome after 2 years of the treatment. Likewise, 88.3% of the 60 Greek cases became seizure free by 1 or 2 years after seizure onset (30). In another study, 29 (69%) cases continued to have seizure after 2 years of receiving antiepileptic drugs (31). Notably, it is difficult to conclude whether seizure control was related to antiepileptic drugs or to natural history of the disease since this condition tend to remit at puberty.

In addition to side effects of drugs, ESES is considered as a factor that negatively impacts the cognitive aspects precisely because it interferes with the cognitive functions of sleep including memory-learning process (7). Even after dividing our cases in two groups based on final SWI, monotherapies especially levetiracetam and normal DQ/IQ at seizure onset seemed to relate with good cognitive outcome. Therefore, it seems like cases with normal DQ/IQ at seizure onset are more likely to remain without cognitive problems. However, few cases can end up with abnormal DQ/IQ after treatment as shown by this study. Polytherapies, utilization of sodium valproate plus other drug (s), levetiracetam plus sodium valproate, initial SWI of ≥85%, and the existence of multifocal spikes correlated with poor cognitive outcome. Topiramate (32), zonisamide (33), and phenobarbital (34) have negative side effects on cognitive function, nevertheless, they did not show any association with cognitive decline in our study. Thus, our study suggests that polytherapies, especially inclusion of sodium valproate should be avoided whenever possible and drugs that mitigate ESES should be encouraged more. Functional MRI study indicated that changes of functional brain network caused by the frequent discharges during slow wave sleep are responsible for cognitive deficits in patients with BECTS (35). Thus, initial SWI of ≥85% observed in our cases played a major role too in cognitive deterioration.

Strikingly, one fourth of our cases received polytherapies. About 10.6% of the cases in another Chinese study used multidrug therapies (8). Some cases with BECTS can have uncontrolled seizures, and some of them can progress to epileptic encephalopathy (8), therefore, there is a possibility that some of our cases received polytherapies due to inability to control seizures and/or worsening of EEG while others due to poor drug compliance.

## Conclusions

Monotherapy, especially levetiracetam seems to be a good first-line therapy which can help in normalizing the EEG and preventing cognitive decline. Polytherapy, particularly the administration of sodium valproate seems to relate with poor cognition, therefore, it is recommended to avoid it.

## Limitations

Despite the fact that our study has some strengths, it has some limitations too. It was retrospective single-center thus prone to bias. The choices of drugs were not uniform for all cases, and they changed according to the clinical observation and updates from the literature. All patients received from 1 to 3 drugs which is quite unusual. It was difficult to comment much on why many cases used polytherapies due to the nature of the study. Noteworthy, the efficacy of the therapies on epilepsy should be interpreted with cautions because seizures could disappear due to natural history of the disease and not due to treatment. Future prospective multicenter studies can evaluate the efficacy of levetiracetam in normalizing the EEG and preventing cognitive decline.

## Data Availability Statement

The original contributions generated for this study are included in the article/Supplementary Material, further inquiries can be directed to the corresponding author/s.

## Ethics Statement

The studies involving human participants were reviewed and approved by the Institutional Ethics Committee of Xiangya Hospital, Central South University. Written informed consent to participate in this study was provided by the participants' legal guardian/next of kin.

## Author Contributions

MK, FYa, and LP are the first co-authors who collected and analyzed data, drafted, and wrote the manuscript. BC, EO, DL, and TR assisted in data collection, analysis as well as in preparation of tables and figures. FH, LY, JP, and FYi designed the study, revised the manuscript, and supervised each step involved in the preparation of the manuscript. All co-authors have read and agreed to the content of the manuscript.

## Conflict of Interest

The authors declare that the research was conducted in the absence of any commercial or financial relationships that could be construed as a potential conflict of interest.
