# Supplementary material for: Treatment for the Benign Childhood Epilepsy With Centrotemporal Spikes: A Monocentric Study
Source: Front Neurol. 2021 May 6;12:670958. doi: 10.3389/fneur.2021.670958 (PMC8134665; doi:10.3389/fneur.2021.670958)
Supplement: Supplementary file 1 [file Table_1.DOCX]

**Supplementary Table 1**

Clinical characteristics for the BECTS cases with all information

| **Clinical characteristics** | **Sample size (N) and percentage** |
| --- | --- |
| **Sex** |  |
| Male | 99/195 (50.8%) |
| Female | 96/195 (49.2%) |
| **Other clinical manifestations** |  |
| Impaired motor skills | 1/195 (0.5%) |
| Abnormal behavior | 3/195 (1.5%) |
| Impaired memory | 11/195 (5.6%) |
| Language impairments | 4/195 (2.1%) |
| Learning problems | 16/195 (8.2%) |
| Social problem | 1/195 (0.5%) |
| **DQ/IQ at onset** |  |
| Normal | 166/181 (91.7%) |
| Abnormal | 15/181 (8.3%) |
| **Ages of onsets** |  |
| Mean age of seizure onset | 6.50 ±2.28 SD, Range=0.17-12.83 |
| Mean age of ESES onset | 7.34±2.39, Range=2-16 |
| **Seizure semiology** |  |
| Partial motor seizures | 18/195 (9.2%) |
| Tonic-clonic seizures | 85/195 (43.6%) |
| Complex partial seizures | 84/195 (43.1%) |
| Epileptic falls | 4/195 (2.1%) |
| Absence seizures | 4/195 (2.1%) |
| Febrile seizures | 7/195 (3.6%) |
| **Spike wave index** |  |
| Spike wave index ≥ 85% | 19/185 (10.3%) |
| Spike wave index <85% | 166/185 (89.7%) |
| **Types of spikes** |  |
| Focal spikes | 108/193 (56%) |
| Multifocal spikes | 18/193 (9.3%) |
| Localized spikes | 161/193 (83.4%) |
| Generalized spikes | 25/193 (13%) |
| **Lateralization of spikes** |  |
| Rolandic | 138/194 (71.1%) |
| Bilateral rolandic | 87/194 (44.8%) |
| Unilateral rolandic | 51/194 (26.3%) |
| Right rolandic only | 26/194 (13.4%) |
| Left rolandic only | 25/194 (12.9%) |
| **Etiologies** |  |
| Known etiology | 31/195 (15.9%) |
| Unknown etiology | 164/195 (84.1%) |
| **Classification of etiology** |  |
| Structural etiology | 28/195 (14.5%) |
| **Brain MRI results** |  |
| Normal | 154/195 (79%) |
| Abnormal | 41/195 (21%) |
| **Treatment strategies** |  |
| Monotherapies | 97/195 (49.7%) |
| Duotherapies | 47/195 (24.1%) |
| Polytherapies | 53/195 (27.2%) |
| **Drugs** |  |
| Levetiracetam plus other drug (s) | 148/195 (75.9%) |
| Levetiracetam | 64/195 (32.8%) |
| Sodium valproate plus other drug (s) | 61/195 (31.3%) |
| Sodium valproate | 10/195 (5.1%) |
| Topiramate plus other drug (s) | 7/195 (3.56%) |
| Zonisamide plus other drug (s) | 1/195 (0.5%) |
| Phenobarbital plus other drug (s) | 1/195 (0.5%) |
| Nitrazepam plus other drug (s) | 16/195 (8.2%) |
| Lamotrigine plus other drug (s) | 12/195 (6.2%) |
| Oxcarbazepine | 14/195 (7.2%) |
| Combined levetiracetam and oxcarbazepine | 25/195 (12.8%) |
| Combined levetiracetam and nitrazepam | 40/195 (20.5%) |
| Combined levetiracetam and sodium valproate | 41/195 (21%) |
| Benzodiazepines | 56/195 (28.7%) |
| AEDs plus steroids | 24/195 (12.3%) |
| **Treatment outcome after 2 years of treatment** |  |
| Seizure free | 116/186 (62.4%) |
| ≥ 50% reduction of seizures | 54/186 (29%) |
| <50% reduction of seizures or same | 28/186 (15.1%) |
| Good prognosis (seizure free and ≥50% reduction) | 132/186 (71%) |
| Disappearance of SWI | 21/182 (11.5%) |
| Improved SWI (disappearance plus ≥50% reduction) | 29/182 (15.9%) |
| No EEG response or became worse | 82/182 (45.1%) |
| Increased SWI | 20/182 (11%) |
| **DQ/IQ after two years of follow up** |  |
| Normal | 166/181 (91.7%) |
| Abnormal | 15/181 (8.3%) |

**Abbreviations**: AEDs: antiepileptic drugs, ADHD: attention deficit hyperactive disorder, BECTS: benign childhood epilepsy with centrotemporal spikes, EEG: electroencephalograph, ESES: electrical status epilepticus in sleep, ID: intellectual disability, MRI: magnetic resonance imaging, SWI: spike wave index.
