# Supplementary material for: Treatment for the Benign Childhood Epilepsy With Centrotemporal Spikes: A Monocentric Study
Source: Front Neurol. 2021 May 6;12:670958. doi: 10.3389/fneur.2021.670958 (PMC8134665; doi:10.3389/fneur.2021.670958)
Supplement: Supplementary file 2 [file Table_2.DOCX]

**Supplementary Table 2: Determinants of positive seizure outcome**

| **Variable** | **Good seizure outcome** | **Poor seizure outcome** | **Overall** | **P-value** |
| --- | --- | --- | --- | --- |
| Prematurity | 4/132 (3 %) | 0/54 (0%) | 4/186 (2.2%) | 0.325 |
| Hypoxic ischemic encephalopathy | 1/132 (0.8%) | 0/54 (0%)) | 1/186 (0.5%) | 0.710 |
| Partial motor seizures | 15/132 (11.4%) | 3/54 (5.6%) | 18/186 (9.7%) | 0.283 |
| Tonic-clonic seizures | 54/132 (40.9%) | 24/54 (44.4%) | 78/186 (41.9%) | 0.744 |
| Complex partial seizures | 60/132 (45.5%) | 22/54 (40.7%) | 82/186 (44.1%) | 0.627 |
| **Epileptic falls** | **1/132 (0.8%)** | **3/54 (5.6%)** | **4/186 (2.2%)** | **0.074** |
| Absence seizures | 4/132 (3%) | 0/54 (0%) | 4/186 (2.2%) | 0.325 |
| Febrile seizures | 7/132 (5.3%) | 0/54 (0%) | 7/186 (3.8%) | 0.109 |
| Impaired motor skills | 1/132 (0.8 %) | 0/54 (0%) | 1/186 (0.5%) | 1.000 |
| Abnormal behavior | 2/132 (1.5%) | 1/54 (1.9%) | 3/186 (1.6%) | 1.000 |
| Impaired memory | 8/132 (6.1%) | 3/54 (5.6%) | 11/186 (5.9%) | 1.000 |
| **Speech delay** | **1/132 (0.8%)** | **3/54 (5.6%)** | **4/186 (2.2%)** | **0.074** |
| Learning problem | 12/132 (9.1%) | 3/54 (5.6%) | 15/186 (8.1%) | 0.559 |
| Social problem | 1/132 (3.4%) | 0/54 (0%) | 1/186 (0.5%) | 1.000 |
| Lack of attention | 6/132 (4.5%) | 2/54 (3.7%) | 8/186 (4.3%) | 1.000 |
| Rolandic origin | 94/132 (71.2%) | 38/53 (71.7%) | 132/185 (71.4%) | 1.000 |
| Bilateral rolandic origin | 62/132 (47.0%) | 22/53 (41.5%) | 84/185 (45.4%) | 0.518 |
| Unilateral rolandic origin | 32/132 (24.2%) | 16/53 (30.2%) | 48/185 (25.9%) | 0.459 |
| Right rolandic origin | 16/132 (12.1%) | 9/53 (17%) | 25/185 (13.5%) | 0.475 |
| Left rolandic origin | 16/132 (12.1%) | 7/53 (13.2%) | 23/185 (12.4%) | 0.810 |
| Focal spikes | 71/132 (53.8%) | 30/52 (57.7%) | 101/184 (54.9%) | 0.742 |
| **Multifocal spikes** | **9/132 (6.8%)** | **8/52 (15.4%)** | **17/184 (9.2%)** | **0.090** |
| Localized spikes | 114/132 (86.4%) | 40/52 (76.9%) | 154/184 (83.7%) | 0.126 |
| Generalized spikes | 16/132 (12.1%) | 7/52 (13.5%) | 23/184 (12.5%) | 0.807 |
| Abnormal MRI | 26/132 (19.7%) | 10/54 (18.5%) | 36/186 (19.4%) | 1.000 |
| Monotherapy | 65/132 (49.2%) | 25/54 (46.3%) | 90/186 (48.4%) | 0.749 |
| Duotherapy | 32/132 (24.2%) | 13/54 (24.1%) | 45/186 (24.2%) | 1.000 |
| Polytherapy | 37/132 (28%) | 16/54 (29.6%) | 53/186 (28.5%) | 0.859 |
| Levetiracetam plus others | 107/132 (81.1%) | 41/54 (75.9%) | 148/186 (79.6%) | 0.430 |
| Topiramate plus others | 3/132 (2.3%) | 4/54 (7.4%) | 7/186 (3.8%) | 0.196 |
| Zonisamide plus others | 1/132 (0.8%) | 0/54 (0%) | 1/186 (0.5%) | 1.000 |
| Phenobarbital plus others | 1/132 (0.8%) | 0/54 (0%) | 1/186 (0.5%) | 1.000 |
| Nitrazepam plus others | 9/132 (6.8%) | 7/54 (13%) | 16/186 (8.6%) | 0.247 |
| Lamotrigine plus others | 7/132 (5.3%) | 5/54 (9.3%) | 12/186 (6.5%) | 0.334 |
| Levetiracetam | 52/132 (39.4%) | 18/54 (33.3%) | 70/186 (37.6%) | 0.506 |
| Sodium valproate plus others | 43/132 (32.6%) | 17/54 (31.5%) | 60/186 (32.3%) | 1.000 |
| **Sodium valproate** | **4/132 (3.0%)** | **6/54 (11.1%)** | **10/186 (5.4%)** | **0.066** |
| Oxcarbazepine | 8/132 (6.1%) | 3/54 (5.6%) | 11/186 (5.9%) | 1.000 |
| Benzodiazepines plus antiepileptic drugs | 37/132 (28%) | 19/54 (35.2%) | 56/186 (30.1%) | 0.380 |
| Antiepileptic drugs plus steroids | 19/132 (14.4%) | 5/54 (9.3%) | 24/186 (12.9%) | 0.471 |
| Levetiracetam and nitrazepam | 26/132 (19.7%) | 14/54 (25.9%) | 40/186 (21.5%) | 0.432 |
| Levetiracetam and sodium valproate | 31/132 (23.5%) | 9/54 (16.7%) | 40/186 (21.5%) | 0.334 |
| Spike wave index ≥85% | 13/131 (9.9%) | 6/54 (11.1%) | 19/185 (10.3%) | 0.794 |
| Known etiology | 25/132 (18.9%) | 6/54 (11.1%) | 31/186 (16.7%) | 0.278 |
| Increased spike wave index | 11/130 (8.5%) | 9/52 (17.3%) | 20/182 (11.0%) | 0.114 |
| No changes in spike waves or increased spike wave index | 54/130 (41.5%) | 28/52 (53.8%) | 82/182 (45.1%) | 0.141 |

**Abbreviations**: AEDs: antiepileptic drugs, MRI: magnetic resonance imaging
