# Supplementary material for: Treatment for the Benign Childhood Epilepsy With Centrotemporal Spikes: A Monocentric Study
Source: Front Neurol. 2021 May 6;12:670958. doi: 10.3389/fneur.2021.670958 (PMC8134665; doi:10.3389/fneur.2021.670958)
Supplement: Supplementary file 3 [file Table_3.DOCX]

**Supplementary Table 3**

**Determinants of positive improvement of spike wave index**

| **Variable** | **Good improvement of SWI** | **Poor improvement of SWI** | **Overall** | **P-value** |
| --- | --- | --- | --- | --- |
| Known etiology | 5/29 (17.2%) | 26/153 (17%) | 31/182 (17%) | 1.000 |
| Prematurity | 1/29 (3.4%) | 3/153(2%) | 4/182 (2.2%) | 0.504 |
| Hypoxic ischemic encephalopathy | 0/29 (0.0%) | 1/153 (0.7%) | 1/182 (0.5%) | 1.000 |
| Partial motor seizures | 2/29 (6.9%) | 16/153 (10.5%) | 18/182 (9.9%) | 0.742 |
| Tonic-clonic seizures | 9/29 (31%) | 65/153 (42.5%) | 74/182 (40.7%) | 0.305 |
| Complex partial seizures | 15/29 (51.7%) | 66/153 (43.1%) | 81/182 (44.5%) | 0.421 |
| Epileptic falls | 1/29 (3.4%) | 3/153 (2.0%) | 4/182 (2.2%) | 0.504 |
| Absence seizures | 1/29 (3.4%) | 3/153 (2.0%) | 4/182 (2.2%) | 0.504 |
| Febrile seizures | 0/29 (0%) | 7/153 (4.6%) | 7/182 (3.8%) | 0.599 |
| Impaired moto skills | 0/29 (0%) | 1/153 (0.7%) | 1/182 (0.5%) | 1.000 |
| Abnormal behavior | 1/29 (3.4%) | 2/153 (1.3%) | 3/182 (1.6%) | 0.408 |
| Impaired memory | 3/29 (10.3%) | 8/153 (5.2%) | 11/182 (6%) | 0.386 |
| Speech problems | 0/29 (0%) | 4/153 (2.6%) | 4/182 (2.2%) | 1.000 |
| Learning problems | 1/29 (3.4%) | 14/153 (9.2%) | 15/182 (8.2%) | 0.472 |
| Social problems | 0/29 (0%) | 1/153 (0.7%) | 1/182 (0.5%) | 1.000 |
| Lack of attention | 3/29 (10.3%) | 5/153 (3.3%) | 8/182 (4.4%) | 0.117 |
| Rolandic spikes | 19/29 (65.5%) | 110/152 (72.4%) | 129/181 (71.3%) | 0.504 |
| Bilateral rolandic spikes | 10/29 (34.5%) | 73/152 (48%) | 83/181(45.9%) | 0.224 |
| Unilateral rolandic origin | 9/29 (31%) | 37/152 (24.3%) | 46/181 (25.4%) | 0.487 |
| Right rolandic origin only | 7/29 (24.1%) | 16/152 (10.5%) | 23/181 (12.7%) | 0.064 |
| Left rolandic origin only | 2/29 (6.9%) | 21/152 (13.8%) | 23/181 (12.7%) | 0.541 |
| **Focal spikes** | **10/29 (34.5%)** | **88/151 (58.3%)** | **98/180 (54.4%)** | **0.025** |
| Multifocal spikes | 3/29 (10.3%) | 14/151(9.3%) | 17/180 (9.4%) | 0.740 |
| **Localized spikes** | **20/29 (69%)** | **130/151 (86.1%)** | **150/180 (83.3%)** | **0.031** |
| Generalized spikes | 6/29 (20.7%) | 17/151 (11.3%) | 23/180 (12.8%) | 0.219 |
| Abnormal MRI | 4/29 (13.8%) | 30/153 (19.6%) | 34/182 (18.7%) | 0.607 |
| Monotherapy | 13/29 (44.8%) | 73/153 (47.7%) | 86/182 (47.3%) | 0.841 |
| Duotherapy | 5/29 (17.2%) | 40/153 (26.1%) | 45/182 (24.7%) | 0.358 |
| Polytherapy | 12/29 (41.4%) | 41/153 (26.8%) | 53/182 (29.1%) | 0.123 |
| Levetiracetam plus others | 24/29 (82.8%) | 121/153 (79.1%) | 145/182 (79.7%) | 0.804 |
| Levetiractem | 10/29 (34.5%) | 57/153 (37.3%) | 67/182 (36.8%) | 0.836 |
| **Sodium valproate** | **15/29 (51.7%)** | **45/153 (29.4%)** | **60/182 (33%)** | **0.030** |
| Topiramate | 2/29 (6.9%) | 5/153 (3.3%) | 7/182 (3.8%) | 0.309 |
| Zonisamide plus others | 0/29 (0%) | 1/153 (0.7%) | 1/182 (0.5%) | 1.000 |
| Phenobarbital plus others | 0/29 (0%) | 1/153 (0.7%) | 1/182 (0.5%) | 1.000 |
| Nitrazepam plus others | 3/29 (10.3%) | 13/153 (8.5%) | 16/182 (8.8%) | 0.723 |
| Sodium valproate | 1/29 (3.4%) | 9/153 (5.9%) | 10/182 (5.5%) | 1.000 |
| **Variable** | **Good improvement of SWI** | **Poor improvement of SWI** | **Overall** | **P-value** |
| Oxcarbazepine | 1/29 (3.4%) | 9/153 (5.9%) | 10/182 (5.5%) | 1.000 |
| Benzodiazepines plus antiepileptic drugs | 10/29 (34.5%) | 46/153 (30.1%) | 56/182 (30.8%) | 0.664 |
| Levetiracetam plus nitrazepam | 7/29 (24.1%) | 33/153 (21.6%) | 40/182 (22%) | 0.808 |
| **Levetiracetam plus sodium valproate** | **12/29 (41.4%)** | **28/153 (18.3%)** | **40/182 (22%)** | **0.012** |
| Antiepileptic drugs and steroids | 5/29 (17.2%) | 19/153 (12.4%) | 24/182 (13.2%) | 0.548 |

**Abbreviations**: AEDs: antiepileptic drugs, MRI: magnetic resonance imaging, SWI: spike wave index.
