# Supplementary material for: Treatment for the Benign Childhood Epilepsy With Centrotemporal Spikes: A Monocentric Study
Source: Front Neurol. 2021 May 6;12:670958. doi: 10.3389/fneur.2021.670958 (PMC8134665; doi:10.3389/fneur.2021.670958)
Supplement: Supplementary file 4 [file Table_4.DOCX]

**Supplementary Table 4: Cognitive outcome after 2 years of treatment for the cases with 0-50% SWI at the last follow up**

| **Variable** | **Good cognitive outcome** | **Poor cognitive outcome** | **Overall** | **P-value** |
| --- | --- | --- | --- | --- |
| Seizures onset age ≤ 4 years | 12/78 (15.4%) | 1/5 (20%) | 13/83 (15.7%) | 0.583 |
| ESES onset age ≤ 4 years | 7/78 (9%) | 1/5 (20%) | 8/83 (9.6%) | 0.406 |
| Rolandic origin | 58/78 (74.4%) | 4/5 (80%) | 62/83 (74.7%) | 1.000 |
| Bilateral rolandic origin | 34/78 (43.6%) | 3/5 (60%) | 37/83 (44.6%) | 0.652 |
| Unilateral rolandic origin | 24/78 (30.8%) | 1/5 (20%) | 25/83 (30.1%) | 1.000 |
| Right rolandic origin | 14/78 (17.9%) | 0/5 (0%) | 14/83 (16.9%) | 0.583 |
| Left rolandic origin | 10/78 (12.8%) | 1/5 (20%) | 11/83 (13.3%) | 0.518 |
| Focal spikes | 43/77 (55.8%) | 2/5 (40%) | 45/82 (54.9%) | 0.654 |
| Multifocal spikes | 5/77 (6.5%) | 1/5 (20%) | 6/82 (7.3%) | 0.323 |
| Localized spikes | 66/77 (85.7%) | 4/5 (80%) | 70/82 (85.4%) | 0.556 |
| Generalized spikes | 8/77 (10.4%) | 1/5 (20%) | 9/82 (11%) | 0.450 |
| Abnormal MRI | 14/78 (17.9%) | 1/5 (20%) | 15/83 (18.1%) | 1.000 |
| Monotherapy | 43/78 (55.1%) | 1/5 (20%) | 44/83 (53%) | 0.182 |
| Duotherapy | 20/78 (25.6%) | 3/5 (60%) | 23/83 (27.7%) | 0.127 |
| Polytherapy | 16/78 (20.5%) | 1/5 (20%) | 17/83 (20.5%) | 1.000 |
| Levetiracetam plus other drugs | 62/78 (79.5%) | 3/5 (60%) | 65/83 (78.3%) | 0.296 |
| **Levetiracetam** | **33/78 (42.3%)** | **0/5 (0%)** | **33/83 (39.8%)** | **0.152** |
| **Sodium valproate plus other drugs** | **21/78 (26.9%)** | **4/5 (80%)** | **25/83 (30.1%)** | **0.027** |
| Sodium valproate | 2/78 (2.6%) | 1/5 (20%) | 3/83 (3.6%) | 0.172 |
| Oxcarbazepine | 8/78 (10.3%) | 0/5 (0%) | 8/83 (9.6%) | 1.000 |
| Benzodiazepines plus antiepileptic drugs | 23/78 (29.5%) | 1/5 (20%) | 24/83 (28.9%) | 1.000 |
| Topiramate plus other drugs | 3/78 (3.8%) | 0/5 (0%) | 3/83 (3.6%) | 1.000 |
| Nitrazepam plus other drugs | 7/78 (9%) | 0/5 (0%) | 7/83 (8.4%) | 1.000 |
| Lamotrigine plus other drugs | 3/78 (3.8%) | 1/5 (20%) | 4/83 (4.8%) | 0.224 |
| Levetiracetam plus oxcarbazepine | 8/78 (10.3%) | 0/5 (0%) | 8/83 (9.6%) | 1.000 |
| Levetiracetam plus nitrazepam | 17/78 (21.8%) | 0/5 (0%) | 17/83 (20.5%) | 0.578 |
| **Levetiracetam plus sodium valproate** | **14/78 (17.9%)** | **3/5 (60%)** | **17/83 (20.5%)** | **0.056** |
| Sodium valproate plus oxcarbazepine | 8/78 (10.3%) | 0/5 (0%) | 8/83 (9.6%) | 1.000 |
| Antiepileptic drugs and steroids | 4/78 (5.1%) | 0/5 (0%) | 4/83 (4.8%) | 1.000 |
| Seizure free | 52/77 (67.5%) | 3/5 (60%) | 55/82 (67.1%) | 1.000 |
| ≥50% seizure frequency reduction | 18/77 (23.4%) | 2/5 (40%) | 20/82 (24.4%) | 0.591 |
| <50% seizure frequency reduction or no improvement | 13/77 (16.9%) | 0/5 (0%) | 13/82 (15.9%) | 1.000 |
| Seizure free plus ≥50% seizure frequency reduction | 59/77 (76.6%) | 5/5 (100%) | 64/82 (78%) | 0.581 |
| **Normal DQ/IQ at seizure onset** | **74/78 (94.9%)** | **0/5 (0%)** | **74/83 (89.2%)** | **0.000** |

**Abbreviations**: AEDs: antiepileptic drugs, BECTS: benign childhood epilepsy with centrotemporal spikes, MRI: magnetic resonance imaging, SWI: spike wave index.
