# Supplementary material for: Treatment for the Benign Childhood Epilepsy With Centrotemporal Spikes: A Monocentric Study
Source: Front Neurol. 2021 May 6;12:670958. doi: 10.3389/fneur.2021.670958 (PMC8134665; doi:10.3389/fneur.2021.670958)
Supplement: Supplementary file 5 [file Table_5.DOCX]

**Supplementary Table 5: Cognitive outcome after 2 years of treatment for the cases with > 50% SWI at the last follow up**

| **Variable** | **Good cognitive outcome** | **Poor cognitive outcome** | **Overall** | **P-value** |
| --- | --- | --- | --- | --- |
| Seizures onset age ≤ 4 years | 8/82 (9.8%) | 2/10 (20%) | 10/92 (10.9%) | 0.297 |
| ESES onset age ≤ 4 years | 9/82 (11%) | 2/10 (20%) | 11/92 (12%) | 0.342 |
| Rolandic origin | 58/81 (71.6%) | 8/10 (80%) | 66/91 (72.5%) | 0.721 |
| Bilateral rolandic origin | 40/81 (49.4%) | 4/10 (40%) | 44/91 (48.4%) | 0.741 |
| Unilateral rolandic origin | 18/81 (22.2%) | 4/10 (40%) | 22/91 (24.2%) | 0.247 |
| Right rolandic origin | 10/81 (12.3%) | 3/10 (30%) | 13/91 (14.3%) | 0.151 |
| Left rolandic origin | 8/81 (9.9%) | 1/10 (10%) | 9/91 (9.9%) | 1.000 |
| Focal spikes | 46/82 (56.1%) | 6/10 (60%) | 52/92 (56.5%) | 1.000 |
| **Multifocal spikes** | **8/82 (9.8%)** | **3/7 (30%)** | **11/92 (12%)** | **0.096** |
| Localized spikes | 66/82 (80.5%) | 9/10 (90%) | 75/92 (81.5%) | 0.682 |
| Generalized spikes | 12/82 (14.6%) | 1/10 (10%) | 13/92 (14.1%) | 1.000 |
| Abnormal MRI | 16/82 (19.5%) | 3/10 (30%) | 19/92 (20.7%) | 0.425 |
| **Monotherapy** | **35/82 (42.7%)** | 3/10 (30%) | 38/92 (41.3%) | 0.515 |
| Duotherapy | **18/82 (22%)** | 1/10 (10%) | 19/92 (20.7%) | 0.681 |
| **Polytherapy** | **29/82 (35.4%)** | **6/10 (60%)** | 35/92 (38%) | 0.172 |
| Levetiracetam plus other drugs | 64/82 (78%) | 8/10 (80%) | 72/92 (78.3%) | 1.000 |
| **Levetiracetam** | **28/82 (34.1%)** | 2/10 (20%) | 30/92 (32.6%) | 0.489 |
| **Sodium valproate plus other drugs** | **26/82 (31.7%)** | **7/10 (70%)** | **33/92 (35.9%)** | **0.032** |
| Sodium valproate | 6/82 (7.3%) | 1/10 (10%) | 7/92 (7.6%) | 0.566 |
| Oxcarbazepine | 5/82 (6.1%) | 0/10 (0%) | 5/92 (5.4%) | 1.000 |
| Benzodiazepines plus other antiepileptic drugs | 25/82 (30.5%) | 5/10 (50%) | 30/92 (32.6%) | 0.285 |
| Topiramate plus other drugs | 3/82 (3.7%) | 0/10 (0%) | 3/92 (3.3%) | 1.000 |
| Zonisamide plus other drugs | 1/82 (1.2%) | 0/10 (0%) | 1/92 (1.1%) | 1.000 |
| Phenobarbital plus other drugs | 1/82 (1.2%) | 0/10 (0%) | 1/92 (1.1%) | 1.000 |
| Nitrazepam plus other drugs | 11/82 (13.4%) | 0/10 (0%) | 11/92 (12%) | 0.602 |
| Lamotrigine plus other drugs | 7/82 (8.5%) | 2/10 (20%) | 9/92 (9.8%) | 0.252 |
| Levetiracetam plus oxcarbazepine | 13/82 (15.9%) | 3/10 (30%) | 16/92 (17.4%) | 0. 370 |
| Levetiracetam plus nitrazepam | 19/82 (23.2%) | 4/10 (40%) | 23/92 (25%) | 0.261 |
| **Levetiracetam plus sodium valproate** | **17/82 (20.7%)** | **5/10 (50%)** | **22/92 (23.9%)** | **0.055** |
| Sodium valproate and oxcarbazepine | 8/82 (9.8%) | 3/10 (30%) | 11/92 (12%) | 0.096 |
| Antiepileptic drugs and steroids | 16/82 (19.5%) | 3/10 (30%) | 19/92 (20.7%) | 0.425 |
| Seizure free | 45/77 (58.4%) | 7/10 (70%) | 52/87 (59.8%) | 0.734 |
| **≥50% seizure reduction** | **30/77 (39%)** | **0/10 (0%)** | **30/87 (34.5%)** | **0.013** |
| <50% seizure reduction or no improvement | 12/77 (15.6%) | 2/10 (20%) | 14/87 (16.1%) | 0.660 |
| Seizure free plus ≥50% seizure frequency reduction | 48/77 (62.3%) | 7/10 (70%) | 55/87 (63.2%) | 0.739 |
| **≥ 85% initial SWI** | **8/76 (10.5%)** | **5/10 (50%)** | **13/86 (15.1%)** | **0.006** |
| **Normal DQ/IQ at seizure onset** | **79/80 (98.8%)** | **5/10 (50%)** | **84/90 (93.3%)** | **0.000** |

**Abbreviations**: AEDs: antiepileptic drugs, BECTS: benign childhood epilepsy with centrotemporal spikes, MRI: magnetic resonance imaging, SWI: spike wave index.
